# Iron-Containing Alcohol Dehydrogenase from Hyperthermophiles

**DOI:** 10.3390/biotech15010006

**Published:** 2026-01-15

**Authors:** Ching Tse, Kesen Ma

**Affiliations:** Department of Biology, University of Waterloo, Waterloo, ON N2L 3G1, Canada; ching.tse@uwaterloo.ca

**Keywords:** alcohol dehydrogenases, hyperthermophiles, thermostability, iron-containing dehydrogenases, biocatalysts

## Abstract

Iron-containing alcohol dehydrogenases (Fe-ADHs) from hyperthermophiles represent a distinct class of oxidoreductases characterized by exceptional thermostability, catalytic versatility, and unique metal-dependent properties. Despite considerable sequence diversity, Fe-ADHs share conserved motifs and a two-domain architecture essential for iron coordination and NAD(P)H cofactor binding. Physiologically, these enzymes are predicted to function primarily in aldehyde detoxification and redox homeostasis, with some also participating in fermentative alcohol production. Their remarkable stability and catalytic efficiency highlight their potential as robust biocatalysts for high-temperature industrial bioprocesses. This review presents a comprehensive comparative analysis of the biophysical, biochemical, and kinetic properties of Fe-ADHs, focusing on their thermostability, metal ion specificity, and catalytic mechanisms, as well as highlighting their potential for industrial biocatalytic applications.

## 1. Introduction

Alcohol dehydrogenases (ADHs) are members of the oxidoreductase family that catalyze the reversible conversion of alcohols to aldehydes or ketones, typically coupled to electron carriers such as NAD^+^/NADP^+^, pyrroloquinoline quinone, flavin adenine dinucleotide, heme, or cofactor F420 [1,2,3,4,5]. Their wide distribution across bacteria, archaea, and eukarya highlights their fundamental roles in cellular metabolism, including both primary pathways such as energy conservation and secondary processes like detoxification [4,6,7]. Decades of research have revealed extensive diversity in ADH structure, cofactor specificity, and catalytic properties, making them attractive targets for biochemical and industrial studies [3,4].

Within the NAD^+^-dependent ADHs, three main types of ADHs are classified based on sequence, molecular size, and metal content. Type I, or short-chain ADHs, are typically around 250 amino acid residues per chain and generally lack metals [8,9]. Type II, also known as medium-chain or zinc-containing ADHs, are about 370 amino acid residues per chain and usually form dimers in eukaryotes or tetramers in prokaryotes [10,11]. Type III alcohol dehydrogenases (ADHs), also referred to as long-chain or iron-dependent ADHs (Fe-ADHs), are composed of approximately 380 to 900 amino acid residues per subunit and shared only limited sequence homology with other ADH families, underscoring their evolutionary divergence [12,13,14,15].

Fe-ADHs represent the most recently identified ADH class, establishing a novel lineage distinct from the previously characterized type I and II ADHs [15]. Fe-ADHs are generally classified as either iron-activated, requiring an iron ion for catalytic activity, or iron-containing, in which the iron atom remains associated with the purified enzyme [16,17,18,19]. The discovery of Fe-ADHs began with the identification of an iron-activated ADH in *Zymomonas mobilis*, followed by the characterization of homologous proteins with diverse enzymatic activities, including butanol dehydrogenase (BdhA and BdhB) from *Clostridium acetobutylicum* and 1,3-propanediol dehydrogenase (DhaT) from *Klebsiella pneumoniae* [12,20,21]. Beyond these mesophilic representatives, homologs of Fe-ADHs have also been identified and characterized in hyperthermophilic archaea and bacteria, organisms that grow optimally at temperatures exceeding 80 °C [22]. Enzymes from these extremophiles typically exhibited remarkable thermophilicity and thermostability, along with enhanced resistance to chemical denaturants [23,24]. These properties render hyperthermophilic Fe-ADHs particularly attractive as biocatalysts for high-temperature industrial processes, underscoring their biochemical diversity and considerable biotechnological potential.

To date, native or recombinant Fe-ADHs have been characterized from nine hyperthermophiles, including thermophilic archaea such as *Thermococcus litoralis* [19,25], *Thermococcus paralvinellae* ES-1 [25,26,27], *Thermococcus zilligii* AN1 [28,29], *Thermococcus hydrothermalis* [30], *Thermococcus barophilus* (Tba ADH_547_ and Tba ADH_641_) [31,32], *Hyperthermus butylicus* [33] and *Pyrococcus horikoshii* OT3 [34], as well as from thermophilic bacteria *Pseudothermotoga hypogea* [35,36] and *Thermotoga neapolitana* (Fe-AAdh) [37]. In these organisms, Fe-ADHs typically catalyze both alcohol oxidation and aldehyde or ketone reduction at extreme temperatures, utilizing NAD(P)H as a cofactor [19,25,26,27,28,29,30,31,32,33,34,35,36,37]. These enzymes provide a comprehensive framework for examining the relationship between structure, thermostability, and catalytic efficiency under extreme temperature conditions.

This review aims to provide a comparative analysis of the biophysical and biochemical properties, as well as the catalytic mechanisms of Fe-ADHs from hyperthermophiles. We also discuss their physiological roles and highlight their potential applications in pharmaceuticals and biofuel production.

## 2. Biophysical and Biochemical Properties of Fe-ADHs from Hyperthermophiles

The biophysical and biochemical properties of Fe-ADHs from hyperthermophiles are summarized in Table 1. Fe-ADHs of hyperthermophiles are endowed with remarkable thermostability and thermophilicity [19,25,26,27,28,29,30,31,32,33,34,35,36,37]. Given that thermal adaptation and thermostability are defining features of these enzymes, the review begins by examining these properties, along with their size and oligomeric state, as variations in these parameters can influence thermostability [38].

### 2.1. Thermostability and Quaternary Structural Features of Fe-ADHs from Hyperthermophiles

The characterized Fe-ADHs generally exist as oligomers, with subunit molecular masses ranging from approximately 40 to 48 kDa [19,25,26,27,28,29,30,31,32,33,34,35,36,37]. They most often occur in tetramers (α_4_), as seen in *T. litoralis* (48 kDa) [19], *T. paralvinellae* ES-1 (46 kDa) [26] and *T. zilligii* AN1 (46 kD) [28,29], or as dimers (α_2_) in *P. hypogea* (40 kDa) [35,36] and *T. hydrothermalis* (45 kDa), which exists as α_4_ for alcohol oxidation or α_2_ for aldehyde reduction [30].

The thermal stability of proteins is generally influenced by several factors, such as a more hydrophobic core; the substitution of glycine or lysine with arginine to promote the formation of more α-helix structures; the presence of additional charged residues to enhance ionic interactions; and the insertion of proline (Pro) residues or substitutions with Pro in loop regions of proteins [4]. For example, in *T. zilligii* AN1, Fe-ADH increases its thermostability by replacing conserved Pro residues with smaller residues (Ala, Ser, and Thr) to alter the protein conformation [28]. In *T. hydrothermalis*, the proximal conserved Pro and the distal one is replaced by alanine and threonine, respectively [30].

Optimal temperatures for ADH activity typically cluster between 80 °C and 95 °C, with some ADHs exceeding this range [19,25,26,27,28,29,30,31,32,33,34,35,36,37]. *T. paralvinellae* ES-1, *P. horikoshii* OT3, and *P. hypogea* exhibited activity optima above 95 °C [25,26,27,34,35,36], while *H. butylicus* ADH remained highly active above 90 °C [33]. In contrast, *T. barophilus* ADH_547_ and ADH_641_ operated most effectively at slightly lower temperatures (65–80 °C) [31,32]. The oligomeric state of *T. barophilus* ADHs may contribute to their relatively low thermophilicity compared with other Fe-ADHs from hyperthermophiles, warranting further investigation.

Thermostability among Fe-ADHs from hyperthermophiles varied widely [19,25,26,27,28,29,30,31,32,33,34,35,36,37]. *H. butylicus* ADH demonstrated exceptional thermostability, retaining activity at 95 °C with a half-life of about 25 h [33]. *T. paralvinellae* ES-1 ADH and *P. horikoshii* OT3 ADH also exhibited strong resilience to high temperatures, with half-lives of 35 h at 85 °C and around 6 h at 95 °C, respectively [26,27,34]. In contrast, *T. barophilus* ADHs were considerably less thermostable, with half-lives of less than 0.5 h at 90 °C and around 20 min at 70 °C [31,32]. Other Fe-ADHs were moderately thermostable, in general, with half-lives of about 1 h to 2 h at 80 °C to 90 °C [19,28,29,30,35,36,37]. The striking contrast between the highly thermostable ADHs from *T. paralvinellae* ES-1, *P. horikoshii* OT3, and *H. butylicus* and the less thermostable ADHs from *T. barophilus* underscores the influence of ecological adaptation on enzyme thermostability. This pattern aligns with their growth temperature profiles: *T. barophilus* has been shown to exhibit a wide growth temperature range of 48 to 100 °C [43], whereas *T. paralvinellae* ES-1, *P. horikoshii* OT3, and *H. butylicus* have optimal growth temperatures above 90 °C [40,44,45].

### 2.2. Effects of Metal Ions and Oxygen on the Activities of Fe-ADHs from Hyperthermophiles

Iron is essential for the catalytic function of Fe-ADHs, which are either Fe-activated or Fe-containing ADHs [16,17,18,19]. In mesophilic Fe-ADHs, the purified enzymes often lack bound iron; rather, iron must be present in the assay mixture to obtain ADH activities [3]. In contrast, hyperthermophilic ADHs have been shown to contain Fe after purification, and these subtle differences between organisms demonstrate how hyperthermophiles may have evolved distinct mechanisms of metal utilization suited to their environments [19,26,27,28,29,30,31,32,33,34,35,36,37]. Despite this shared Fe-dependence, variation exists in Fe-ion specificity, tolerance to metal substitution, and resistance to oxidative inactivation [19,26,27,28,29,30,31,32,33,34,35,36,37]. These differences suggest evolutionary fine-tuning of metal coordination and redox stability among different hyperthermophiles.

In characterized Fe-ADHs, the iron was present mostly in the ferrous (Fe^2+^) form after purification and was required for catalytic activity [25,35,36]. For example, in *T. litoralis* and *T. paralvinellae* ES-1 ADHs, Fe^2+^ was more readily lost in *T. litoralis*, whereas in *T. paralvinellae* ES-1, both the ferric (Fe^3+^) ion and Fe^2+^ bound tightly to the anaerobically purified enzyme in nearly stoichiometric amounts [25]. Enzymatic activities could not be restored simply by adding Fe^2+^ or Fe^3+^ in the assay mixture under either anaerobic or aerobic conditions, suggesting stringent structural requirements for proper metal incorporation for activity [25]. In the hyperthermophilic bacterium *P. hypogea* ADH, Fe^2+^ was required for catalysis, but Zn^2+^ acted as an inhibitor, which suggests that extraneous Zn^2+^ can replace iron that may be dissociated during oxygen exposure [35,36]. This inhibition effect could be relieved by thiol reductants such as dithiothreitol (DTT), suggesting that competitive metal binding can disrupt activity but is reversible under reducing conditions [35,36]. Full recovery of activity could also be achieved by incubating the ADH with both DTT and Fe^2+^ under anaerobic conditions for about one hour [35,36].

For recombinant *T. barophilus* ADHs, distinct metal preferences have been observed [31,32]. ADH_547_ did not require divalent ions and was optimally activated by Fe^2+^ for aldehyde reduction, but it depended on divalent ions for ethanol oxidation, with Mn^2+^ optimal [31]. In contrast, ADH_641_ had the opposite requirement; it could oxidize ethanol without added divalent ions (though activity was maximal with Fe^2+^), and it required divalent ions, optimally Fe^2+^ or Mg^2+^, to reduce acetaldehyde [32]. Notably, metal substitution studies in *P. horikoshii* OT3 recombinant ADH highlighted functional flexibility [34]. When nickel ion (Ni^2+^) was incorporated into the culture medium, a significantly more active and oxygen-tolerant Ni-binding ADH was produced compared to the Fe-containing ADH [34]. The Ni-ADH could also be reactivated by divalent ions (Mn^2+^, Fe^2+^, Co^2+^, and Ni^2+^), with Ni^2+^ yielding the highest activity toward butanal reduction, even after removal of the original metal ion [34]. In comparison, Fe-containing ADH of *P. horikoshii* OT3 was somewhat irreversibly inactivated when prepared under aerobic conditions [34]. These findings suggest that although Fe^2+^ is the physiological cofactor in the ADHs, alternative divalent cations can modify enzyme activity and stability, likely reflecting potentially adaptive strategies to metal availability in hyperthermophilic environments and highlighting the potential physiological importance of Fe-ADHs in hyperthermophiles [34].

It is not uncommon that the iron-containing enzyme can be reactivated by incubation with iron ions, for example, fumarase in *Bacteroides thetaiotaomicron* was restored to its full activity by Fe^2+^, and aconitase in the same organism was recovered by the treatment of Fe^2+^ and DTT [48]. Metal substitution of group III ADHs has also been reported in the characterization of ADH2 from *Z. mobilis* [49].

Since all Fe-ADHs from hyperthermophiles contain iron, oxygen sensitivity is almost a universal characteristic [19,25,26,27,28,29,30,31,32,33,34,35,36,37]. The inactivation of Fe-containing ADHs may likely be due to the oxidation of Fe^2+^ to Fe^3+^ and the subsequent oxidation of a histidine ligand in the enzyme by Fe^3+^ [50]. Irreversible activity loss may also arise from oxidation of cysteine or other amino acid residues, leading to irreversible structural alterations and enzyme inactivation [50]. Nevertheless, not all hyperthermophilic Fe-ADHs are equally oxygen-sensitive [28,29,34]. The *T. zilligii* AN1 native ADH displayed relative oxygen resistance, and *P. horikoshii* OT3 ADH demonstrated that by replacing Fe^2+^ with Ni^2+^ can provide oxygen tolerance, as Ni^2+^ does not undergo redox cycling as readily as Fe^2+^ or Fe^3+^ [28,29,34]. Moreover, *T. barophilus* recombinant ADHs could be activated by other divalent ions, possibly leading to oxygen insensitivity [31,32]. Collectively, these findings suggest that alternative metal incorporation provides a promising strategy to mitigate the oxygen sensitivity of Fe-ADHs, enhancing their potential for industrial applications.

### 2.3. Substrate Specificity of Fe-ADHs from Hyperthermophiles

Substrate specificity among hyperthermophilic Fe-ADHs is broad, encompassing both aliphatic and aromatic primary alcohols, diols, aldehydes, acetone, and cofactors [19,25,26,27,28,29,30,31,32,33,34,35,36,37]. Comparative analyses across characterized Fe-ADHs reveal both broad and selective substrate preferences that influence catalytic efficiency and potential industrial applications [19,25,26,27,28,29,30,31,32,33,34,35,36,37].

Among the characterized Fe-ADHs from *Thermococcus* species, *T. zilligii* AN1 and *T. barophilius* ADH_641_ favored NADP(H) as the cofactor, while other ADHs used NADP(H) nearly exclusively as the cofactor for oxidation of alcohols and aldehydes or ketones reduction [19,25,26,27,28,29,30,31,32]. *T. litoralis* primarily targeted aliphatic alcohols, showing the highest activity toward 1-hexanol [19]. *T. zilligii* AN1 exhibited the greatest activity toward pentanol among alcohols and with a narrower substrate profile [28,29]. Both ADHs displayed lower apparent *K*_m_ (*K*_m_) values for acetaldehyde reduction than for ethanol oxidation [19,28,29]. In *T. paralvinellae* ES-1, the substrate range expanded beyond aliphatic alcohols to include aromatic and heterocyclic alcohols such as 2-phenylethanol and tryptophol, although catalytic efficiency decreased with increasing molecular size [26,27]. This ADH also displayed strong acetaldehyde reductase activity, favoring acetaldehyde over phenylacetaldehyde, with a higher apparent *V*_max_ value [26,27]. The broad substrate specificity and NADPH-dependent reduction activities suggested that *T. paralvinellae* ES-1 has an unusually flexible active site optimized for redox versatility [26,27]. *T. hydrothermalis* exhibited a diverse substrate recognition among *Thermococcus* enzymes, oxidizing aliphatic alcohols (C_2_–C_8_) as well as cyclic and aromatic substrates, with high catalytic efficiency toward benzyl alcohol and hexanol [30]. It also efficiently reduced benzaldehyde, indicating a well-balanced oxidative and reductive capacity [30]. Two Fe-ADHs characterized from *T. barophilus* showed overlapping yet distinct substrate profiles [31,32]. *T. barophilus* ADH_547_ preferentially oxidized 1-butanol, followed by 1-hexanol and ethanol, whereas *T. barophilus* ADH_641_ exhibited a narrower oxidation range, favoring ethanol, 1-butanol, and then 1-hexanol [31,32]. Both ADHs reduced acetaldehyde, butyraldehyde, caproaldehyde, and acetone, reflecting moderate alcohol and aldehyde redox balance; *T. barophilus* ADH_641_, by contrast, exhibited higher aldehyde reductase efficiency [31,32].

Beyond *Thermococcus*, Fe-ADHs from other hyperthermophiles show comparable versatility [33,34]. *H. butylicus* Fe-ADH catalyzed both primary and secondary alcohol oxidation, with 1-butanol showing the highest activities, and reduced butyraldehyde, acetone, and acetaldehyde [33]. *P. horikoshii* OT3 displayed a broad substrate spectrum, oxidizing both linear (C_2_–C_8_), diols, benzyl alcohol, with the highest activity toward 1-butanol, followed by 1-octanol and 1-heptanol; it also reduced linear aldehydes (C_1_–C_8_) with high NADH-linked efficiency [34].

Among bacterial homologs, *P. hypogea* Fe-ADH displayed broad catalytic breadth, acting on aliphatic alcohols, diols, aldehydes, and methylglyoxal, and showing the highest NADP(H)-dependent activity toward 1-butanol and butyraldehyde, respectively [35]. *T. neapolitana* expressed a bifunctional Fe-AAdh with specificity for acetyl-coenzyme A (acetyl-CoA), acetaldehyde, and ethanol, suggesting a role of this ADH in acetyl-CoA reduction to ethanol production during fermentative metabolism [37].

### 2.4. Optimal pHs for Oxidation and Reduction of Fe-ADHs from Hyperthermophiles

Fe-ADHs from hyperthermophilic archaea and bacteria exhibit optimal catalytic activity under mildly to strongly alkaline conditions for alcohol oxidation, typically ranging from pH 8.0 to 11.0 [19,25,26,27,28,29,30,31,32,33,34,35,36,37]. *T. hydrothermalis* and *P. hypogea* showed the highest pH optima (10.5 and 11.0, respectively), whereas *T. zilligii* AN1 functioned best near neutral pH (6.8–7.0) for oxidation [28,29,30,35,36]. In contrast, aldehyde and ketone reduction generally occurred at lower, near-neutral to slightly acidic pH values, between 5.5 and 8.0 [19,25,26,27,28,29,30,31,32,33,34,35,36,37]. Notably, *H. butylicus* and *P. horikoshii* OT3 displayed the lowest reduction optima (pH 6.0 and 5.5, respectively), indicating pH-dependent modulation of redox activity across these thermostable enzymes [33,34].

### 2.5. Kinetic Properties of Fe-ADHs from Hyperthermophiles

Fe-ADHs from hyperthermophilic archaea and bacteria exhibit considerable diversity in substrate specificity and catalytic turnover, reflecting adaptation to distinct physiological contexts [19,25,26,27,28,29,30,31,32,33,34,35,36,37]. Overall, Fe-ADHs exhibited consistently higher efficiencies in the reduction of aldehydes and ketones than in alcohol oxidation, suggesting a physiological bias toward maintaining redox balance in high-temperature environments [19,25,26,27,28,29,30,31,32,33,34,35,36,37]. This reductive preference also highlights their potential utility as industrial biocatalysts, particularly for the production of alcohols as potential biofuels.

For alcohol oxidation, *K*_m_ values ranged from 0.52 mM for 1-butanol in *P. horikoshii* OT3 to 92 mM for ethanol in *T. barophilus* ADH_547_ [31,34]. In comparison to *Saccharomyces cerevisiae* Adh1, the *K*_m_ values for ethanol range from 17 mM to 24 mM [51]. Turnover rates (*k*_cat_) varied from 0.48 s^−1^ for oxidation of 1-butanol in *P. horikoshii* OT3 to high rates of about 48 s^−1^ for oxidation of butanol in *P. hypogea* and ethanol oxidation in *T. paralvinellae* ES-1 [26,27,35,36]. Catalytic efficiency (*k*_cat_/*K*_m_) further emphasized these contrasts, while *T. barophilus* ADH_547_ only achieved approximately 2.9 s^−1^ M^−1^ for ethanol oxidation, other Fe-ADHs displayed efficiencies exceeding 10^3^ s^−1^ M^−1^ [19,25,26,27,28,29,30,31,32,33,34,35,36,37]. For instance, *T. litoralis* exhibited a relatively modest efficiency of about 2.4 × 10^3^ s^−1^ M^−1^ for ethanol oxidation, *T. paralvinellae* ES-1 demonstrated higher efficiency, such as with ethanol (about 6 × 10^3^ s^−1^ M^−1^), phenylethanol (about 2 × 10^3^ s^−1^ M^−1^) and tryptophol (about 3 × 10^3^ s^−1^ M^−1^), highlighting good catalytic efficiency for a wide range of alcohols, and *T. hydrothermalis* exceeded 10^4^ s^−1^ M^−1^ for benzyl alcohol oxidation [19,26,30]. *P. hypogea* and *H. butylicus* also achieved about 3 × 10^4^ s^−1^ M^−1^ and 3 × 10^3^ s^−1^ M^−1^ with butanol, respectively [33,35].

On the reductive side, in comparison, aldehyde and ketone conversions generally displayed higher catalytic efficiencies [19,25,26,27,28,29,30,31,32,33,34,35,36,37]. For aldehyde reduction, *K*_m_ values ranged from 0.01 mM for benzaldehyde in *T. hydrothermalis* to about 34 mM for acetaldehyde in *T. barophilus* ADH_547_ [30,31]. Kinetics of ketone reduction were measured only in *H. butylicus*, with a *K*_m_ value of 0.68 mM [33]. The *k*_cat_ values of substrates measured spanned from 1.7 s^−1^ (*T. hydrothermalis*, benzaldehyde) to 48 s^−1^ (*P. hypogea*, benzaldehyde). Notably, *T. paralvinellae* ES-1 and *T. hydrothermalis* exhibited strong NADPH-dependent aldehyde reduction, with catalytic efficiencies (*k*_cat_/*K*_m_ values) of approximately 10^5^ s^−1^ M^−1^ for various aldehydes [26,27,30]. *T. barophilus* ADHs displayed relatively lower efficiencies (10^3^ to 10^4^ s^−1^ M^−1^), consistent with their reduced thermostability [31,32]. In contrast, highly thermostable Fe-ADHs, including those from *H. butylicus*, *P. horikoshii* OT3, and *P. hypogea*, showed remarkable specialization for reductive catalysis [33,34,35]. *H. butylicus* achieved 8.1 × 10^3^ s^−1^ M^−1^ for acetone and 1.4 × 10^4^ s^−1^ M^−1^ for butyraldehyde, P. *horikoshii* OT3 showed catalytic efficiencies of 2.8 × 10^3^ s^−1^ M^−1^ for propanal and up to 7.5 × 10^4^ s^−1^ M^−1^ for pentanal in NADH-dependent reduction, and *P. hypogea* efficiently catalyzed butyraldehyde (1.1 × 10^5^ s^−1^ M^−1^) with NADPH as the cofactor [33,34,35]. *T. neapolitana* exhibited moderate turnover, with a *k*_cat_ of about 0.56 s^−1^ for acetyl-CoA and an efficiency of 320 s^−1^ M^−1^, which is consistent with its role in ethanol production with acetaldehyde as the intermediate [37].

These comparisons suggest that, in hyperthermophiles, aldehyde reduction often surpasses alcohol oxidation in catalytic performance. This trend likely reflects a physiological priority for aldehyde detoxification and the maintenance of intracellular redox balance, and it underscores the potential of hyperthermophilic Fe-ADHs as robust biocatalysts for reductive steps in bioalcohol production.

### 2.6. Conserved Motifs and Structural Features of Fe-ADHs from Hyperthermophiles

**Figure 1:**
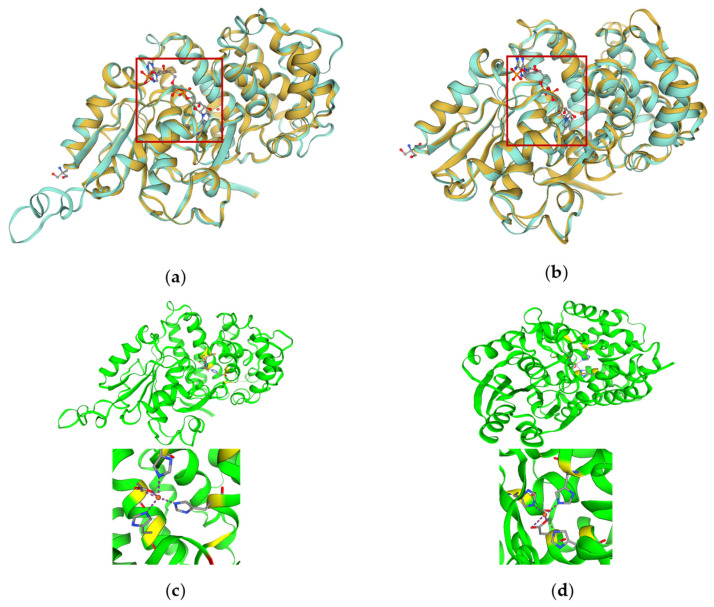
Predicted tertiary structures of the monomer of Fe-ADHs from hyperthermophiles. All 3D structural modeling was run on the Swiss Model server (https://swissmodel.expasy.org (accessed on 31 December 2025)). Superimposition of the predicted 3D tertiary structure of (**a**) *T. paralvinellae* ES-1 ADH monomer (in turquoise) and (**b**) *P. hypogea* ADH monomer (in turquoise) onto the 3D tertiary structure of iron-containing ADH from *Thermotoga maritima* (TM0920; PDB number: 1O2D) (in gold) with the NAD(P)^+^ putative binding sites outlined in red boxes. (**c**) The vertical view of the putative iron-binding site of *T. paralvinellae* ES-1 ADH; amino acid residues in yellow indicate a putative iron-binding site (D209H213H278H293 are predicted to coordinate Fe directly). (**d**) The vertical view of the putative iron-binding site of *P. hypogea* ADH; amino acid residues in yellow indicate a putative iron-binding site (D195H199His268His282 are predicted to coordinate Fe directly).

Despite the considerable biophysical and biochemical diversity of Fe-ADHs from hyperthermophiles, these enzymes share conserved motifs that underpin their catalytic mechanisms [15]. All Fe-ADHs possess two distinct domains separated by a deep cleft, which is an α/β N-terminal domain (domain A) displaying a Rossmann-fold structure that harbors the coenzyme-binding site and a C-terminal α-helical domain (domain B) that contains the Fe-binding site [15]. Both domains are integral to the catalytic mechanism of the enzyme [20,52,53]. Figure 1 illustrates the predicted tertiary structures of Fe-ADHs from hyperthermophiles and the predicted Fe- and cofactor-binding sites.

A hallmark of Fe-ADH is the conserved “GGGS” motif (residues 138 to 141, according to human ADHFE1 numbering), present in all Fe-ADH subfamilies [15]. This motif interacts with the pyrophosphate group of NAD(P)^+^ and forms a loop that connects the β4 strand and the α4 helix [15]. Most Fe-ADHs coordinate Fe^2+^ tetrahedrally through ion-dipole interactions with four conserved residues, namely one aspartate (Asp) and three histidines (His) [15].

Compared to Zn-ADHs, the zinc ions are identified as either structural zinc or catalytic zinc [4]. In general, structural zinc is typically coordinated by four cysteine residues, Cys111, Cys103, Cys97, and Cys100 (numbered according to horse liver ADH), in a tetrahedral manner [54,55]. However, this coordination is not conserved in all hyperthermophilic ADHs, as seen with the replacement of Cys by aspartic acid (Asp) in *Aeropyrum pernix* ADH [56], and by glutamate residues in *Sulfolobus solfataricus* [57]. The catalytic zinc in primary ADHs is most commonly coordinated by four ligands, three of which are Cys48, His68, and Cys174 (again using horse liver ADH numbering) [54]. In secondary ADHs, the C-terminal Cys ligand is replaced by an Asp residue, as observed in those from *Thermoanaerobacter ethanolicus* strain 39E [58] and *Thermoanaerobacter* brockii [59].

**Figure 2:**
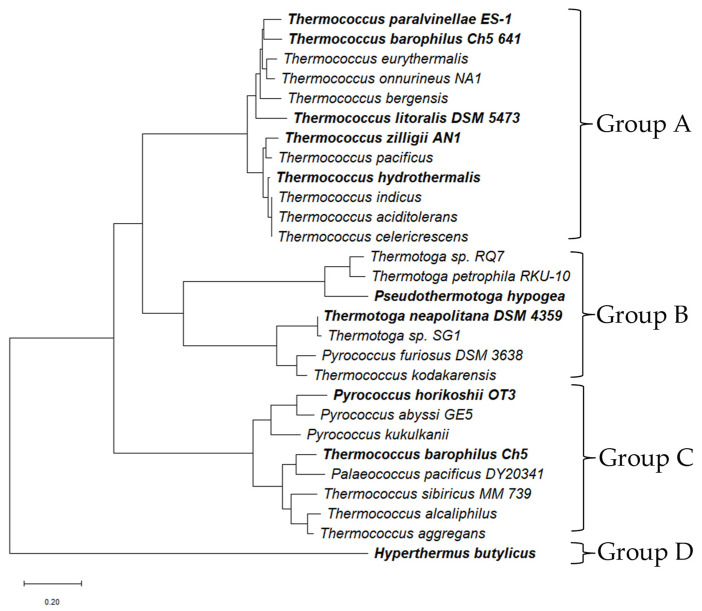
Phylogenetic analysis of iron-containing alcohol dehydrogenases (Fe-ADHs) from hyperthermophiles. The organisms in bold are those from which the Fe-ADHs have been characterized. *Pyrococcus furiosus* DSM 3638 represents iron-containing AdhB, which has not been characterized. Sequences were obtained from the NCBI database, and their accession numbers are provided in Supplementary Table S1.

Phylogenetic and sequence analyses of experimentally characterized Fe-ADHs, along with predicted Fe-ADHs from hyperthermophilic genomes, resolve these ADHs into four distinct clades (Groups A to D), as illustrated in Figure 2. The predicted cofactor-binding and iron-binding motifs of characterized Fe-ADHs from Groups A to D are displayed in Table 2. Hyperthermophilic ADHs in Groups A to C share conserved amino acid residues in similar locations that form key functional motifs, which include a glycine-rich stretch involved in cofactor (NAD(P)H) binding and His residues for catalytic stabilization and the formation of the iron coordination site. In contrast, the Fe-ADH from *H. butylicus* forms a distinct clade (Group D) and exhibits the most divergent predicted cofactor (“GGGXS”) and Fe-binding motifs among analyzed sequences. This divergence suggests that this ADH represents a novel type of Fe-containing ADH among hyperthermophiles [33]. Notably, it is the only ADH that efficiently catalyzes secondary alcohols while exhibiting exceptional thermostability [33].

Sequence alignments among *Thermococcus* species, including characterized ADHs from *T. hydrothermalis*, *T. zilligii* AN1, *T. litoralis*, *T. paralvinellae* ES-1, and *T. barophilus* ADH_641_, clustered within Group A and shared about 75% sequence identity. These ADHs retain the conserved glycine-rich loop and histidine cluster, which are predicted to mediate cofactor and Fe-ion binding, respectively [20,52,53]. The two characterized bacterial Fe-ADHs cluster within Group B, whereas *T. barophilus* ADH_547_ and *P. horikoshii* OT3 form a separate cluster (Group C), reflecting distinct evolutionary relationships. Although these ADHs share similar catalytic motifs, they differ markedly in their biochemical and biophysical properties [19,25,26,27,28,29,30,31,32,33,34,35,36,37].

In *T. barophilus*, two distantly related Fe-ADHs (ADH_547_ in Group C and ADH_641_ in Group A) displayed divergent metal-binding and catalytic characteristics [31,32]. Site-directed mutagenesis identified Asp195 and His262 as essential residues for catalytic activity and for maintaining structural roles in binding divalent ions such as Fe^2+^ in ADH_547_, while His199, His266, and His274 contributed partially but were collectively essential for catalysis [31]. In ADH_641_, the Asp115 residue located within the conserved “GGGSPID” motif was indispensable for both ethanol oxidation and acetaldehyde reduction, which also suggested its central role in Fe^2+^ binding [32]. Additional residues (K118, E159, D190, and E215) were also found to be implicated in maintaining the catalytic geometry and divalent ion interactions [32]. Phylogenetic analysis indicates that these two ADHs belong to different clusters, consistent with their distinct biochemical characteristics and thermostability.

To date, crystal structures of only two Fe-ADHs from hyperthermophiles have been solved, one from the hyperthermophilic bacterium *Thermotoga maritima* (PDB: 1O2D) and another from the hyperthermophilic archaeon *Thermococcus thioreducens* (PDB: 6C75) [60,61]. In *T. maritima* TM0920 (a putative iron-containing 1,3-propanediol dehydrogenase), the Fe^2+^ ion resides deep within the catalytic cleft and is coordinated in a square-pyramidal geometry by Asp189, His193, His256, and His270 in domain B [60]. The NADP^+^-binding site is located between domains A and B, interacting with surrounding residues including the conserved “GGGS” motif in the active site [60]. Similarly, the Fe-ADH in *T. thioreducens* can be divided into two domains, A and B, with the NADP^+^ binding site located in domain A and the Fe^2+^ ion binding site in domain B [61]. Specifically, the Fe^2+^ ion is bound to the second domain by the side chains of Asp193, His197, His260, and His272, which occupy four of the five positions of a trigonal bipyramid around the Fe atom [61]. Also, three loops in domain A form one wall of the tunnel, allowing direct interactions with NADP^+^ [61].

Overall, Fe-ADHs from these hyperthermophiles share conserved iron and cofactor-binding motifs (e.g., “GGGS”, and His/Asp clusters) reflecting a shared mechanistic framework, yet exhibit specific variations in thermostability, oxygen sensitivity, oligomerization, and catalytic properties. These adaptations may correspond to their respective physiological functions and environmental niches in hyperthermophilic ecosystems.

## 3. Physiological Functions of Fe-ADHs in Hyperthermophiles

Fe-ADHs in hyperthermophiles perform diverse yet convergent physiological roles, primarily centered on aldehyde detoxification and alcohol formation, closely tied to the metabolic requirements of life at extreme temperatures. Across the characterized ADHs, a strong catalytic preference for aldehyde reduction over alcohol oxidation was observed, coupled with NAD(P)H dependence, suggesting their role in maintaining redox balance and mitigating aldehyde toxicity [19,25,26,27,28,29,30,31,32,33,34,35,36,37].

In *T. litoralis*, biochemical evidence points to aldehyde reduction as the primary physiological role, as the ADH demonstrated a much lower *K*_m_ value for acetaldehyde (*K*_m_ = 0.40 mM) than for ethanol (*K*_m_ = 11 mM) [19]. In hyperthermophiles, aldehydes are generated in vivo by bifunctional 2-keto acid ferredoxin oxidoreductases, and this ADH likely functions to detoxify the aldehydes produced [25,62]. This interpretation is consistent with the observation that *T. litoralis* does not utilize alcohols as metabolic substrates and its ADH is regarded as a primary ADH [19].

A comparable role is observed in *T. paralvinellae* ES-1, although regulation of its Fe-ADH appears to be tightly linked to elemental sulfur (S^0^) availability during growth on peptide-containing media [25,26,27]. Under the limitation of S^0^, ADH activity markedly increased, accompanied by a fourfold rise in alcohol (ethanol and butanol) production [26]. It has been proposed that S^0^ serves as a terminal electron acceptor, and under the S^0^-limited conditions, when electron acceptors are scarce, the ADH functions to dispose of excess reductant by reducing aldehydes to alcohols during fermentative metabolism, thereby maintaining redox balance [26,27]. Kinetic analyses further support its preference for aldehyde reduction, with *K*_m_ values for aldehydes and NADPH much lower than those for the alcohols examined [26,27]. Its high cellular content in the cell-free extract (up to 10% of cell protein) further implies other important physiological roles [26].

In *T. zilligii* AN1, the ADH likewise displayed a much lower *K*_m_ value for acetaldehyde than for ethanol, again supporting its primary role in aldehyde detoxification [28,29]. Its sequence shares homology with the C-terminal region of bacterial multifunctional oxidoreductases (AdhE proteins from *C. acetobutylicum* and *Escherichia coli*), which combine acetaldehyde and alcohol dehydrogenase activities [20,28,63]. This suggests that archaeal ADHs may represent evolutionary analogues of these bacterial multifunctional oxidoreductases while retaining a reductive physiological bias [20,28,63].

*T. hydrothermalis* offered another variation, in which the recombinant enzyme demonstrated pH-dependent oligomerization [30]. At neutral pH, the enzyme was dimeric and catalytically competent, favoring aldehyde reduction with a *K*_m_ value for benzaldehyde more than tenfold lower than that for benzyl alcohol [30]. At alkaline pH, it formed inactive tetramers [30]. These findings indicate that its physiological role is also aldehyde detoxication, with structural switching providing an additional allosteric regulatory mechanism, similar to that observed in *S. cerevisiae* [30,64].

Distinct specialization is observed in *T. barophilus*, which encodes two Fe-ADHs, ADH_547_ and ADH_641_, sharing only 26% sequence similarity [31,32]. Both enzymes favored aldehyde reduction, potentially contributing to NAD(P)^+^ regeneration during anaerobic glycolysis via the Entner–Doudoroff pathway [31,32]. However, their biochemical properties differ [31,32]. ADH_547_ had a substantially greater catalytic efficiency for aldehyde reduction than for alcohol oxidation, with the *K*_m_ value for acetaldehyde reduction approximately threefold lower than that for ethanol oxidation [31]. By contrast, ADH_641_ was more thermostable in reductive catalysis, displaying a 193-fold greater catalytic efficiency for acetaldehyde reduction than for ethanol oxidation [32]. Such paralogous diversification likely reflects adaptation to the physicochemical conditions of the extremely high-temperature environments in which *T. barophilus* thrives [43].

Other hyperthermophiles reinforce this aldehyde-focused role. In *P. horikoshii* OT3, incorporation of Ni^2+^ into its recombinant ADH generated an ADH with a particularly high turnover (higher *k*_cat_ and lower *K*_m_) for aldehyde reduction compared with alcohol oxidation [34]. Similarly, both recombinant and native *P. hypogea* ADHs exhibited catalytic activities for aldehyde reduction severalfold greater than for alcohol oxidation at pH 8.0, indicating that this ADH efficiently catalyzes aldehyde detoxification near the physiological neutral pH [35,36]. In *H. butylicus*, the ADH had the highest catalytic efficiency for butyraldehyde, catalyzing its reduction to 1-butanol [33]. This suggests an extension of its function beyond aldehyde reduction into primary fermentation pathways, as 1-butanol is a major fermentation product of this archaeon [33,44]. This direct link between Fe-ADH activity and butanol production highlights the potential metabolic integration of these Fe-ADHs in central energy metabolism [33,44].

Finally, *T. neapolitana* provides a striking example of functional divergence within this Fe-ADH family [37]. Its Fe-AAdh is a bifunctional aldehyde/alcohol dehydrogenase, the complete interconversion between acetyl-CoA and ethanol via an acetaldehyde intermediate [37]. This enzyme bridges fermentative pathways, implying a central physiological role of Fe-ADH in energy metabolism and ethanol formation at extremely high temperatures of up to 95 °C, its optimum for oxidation reactions [37]. The Fe-AAdh gene is highly conserved among *Thermotoga* species, and homologous enzymes have been identified in *Pyrococcus furiosus* and *Thermococcus kodakarensis*, suggesting that CoA-dependent aldehyde dehydrogenase and ethanol interconversion constitute a conserved fermentation pathway among hyperthermophiles [37,65]. This mechanism may also form a biochemical framework for potential thermophilic bioethanol production [37].

Taken together, these findings demonstrate that Fe-ADHs in hyperthermophiles function primarily as aldehyde reductases rather than alcohol oxidizers, with physiological roles spanning detoxification, redox homeostasis, and, in *T. neapolitana* and *H. butylicus*, direct participation in ethanol and butanol production [33,37]. Across these hyperthermophiles, most utilize modified Embden–Meyerhof–Parnas and Entner–Doudoroff pathways that generate pyruvate as a central intermediate for amino acid biosynthesis, with acetaldehyde produced as a byproduct; reduction of this aldehyde by ADHs not only prevents toxicity but also recycles reduced cofactors (NAD(P)^+^) [66,67,68,69,70]. The presence of multiple Fe-ADHs within a single hyperthermophile also underscores functional diversification [31,32]. Evolutionary analyses further suggest that archaeal Fe-ADHs share motifs with bacterial multifunctional oxidoreductases such as AdhE, raising the possibility of convergent evolution in aldehyde metabolism [15].

## 4. Industrial Application Potential of Fe-ADHs from Hyperthermophiles

Hyperthermophiles represent promising sources of biocatalysts for industrial biotechnology, particularly in biofuel production and in the synthesis of enantiomerically pure chiral alcohols for industrial or pharmaceutical applications [4,7,71,72]. Their enzymes, including Fe-ADHs, exhibit remarkable thermostability and catalytic activity at temperatures exceeding 80 °C [19,25,26,27,28,29,30,33,34,35,36,37]. These characteristics confer multiple advantages, including elimination of costly cooling steps during fermentation, and increased solubility of hydrophobic substrates, reduced contamination risk, lower viscosity, and decreased end-product inhibition during high-temperature industrial reactions [4,7,71,72].

The application of Fe-ADHs in high-temperature bioethanol or biobutanol production has gained increasing attention, especially those Fe-ADHs capable of catalyzing the reduction of acetaldehyde or butyraldehyde to ethanol or butanol with high efficiency [7,33,37]. For instance, the Fe-AAdh from *T. neopolitana* catalyzes the sequential reduction of acetyl-CoA to ethanol via acetaldehyde through a single bifunctional thermostable ADH, supporting consolidated high-temperature fermentation strategies [37]. Similarly, the native Fe-ADH from *H. butylicus* efficiently catalyzes butyraldehyde reduction to butanol at elevated temperatures [33].

While most Fe-ADHs are oxygen-sensitive, necessitating specialized equipment such as anaerobic chambers and oxygen sensors, their use in industrial applications may result in higher capital and operational costs [73]. However, despite these challenges, the initial step of biofuel production, specifically bioethanol, in biorefineries relies on the anaerobic fermentation of various sugars and starches (classified as first-generation biofuels) or lignocellulosic biomasses (recognized as second-generation biofuels) [74]. This fermentation process requires careful management of anaerobic conditions, which makes Fe-ADHs from hyperthermophiles potentially advantageous in this context. Furthermore, Fe-ADHs derived from hyperthermophiles have demonstrated a remarkable ability to degrade a variety of substrates, including lignocellulosic materials, presenting a valuable avenue for potential biofuel production [4,19,25,26,27,28,29,30,31,32,33,34,35,36,37].

Many hyperthermophilic organisms are capable of alcohol fermentation, and the concentrations of alcohol produced are typically low (in the millimolar range) [4]. However, Fe-ADHs can catalyze reversible reactions under higher alcohol concentrations, which supports their role as essential housekeeping enzymes [75]. Metabolic engineering has also been applied to optimize ethanol production in thermophiles [7]. Overexpression of bifunctional alcohol/aldehyde dehydrogenases in *T. ethanolicus* JW200 resulted in a 40% increase in ethanol yield [76]. In *Thermoanaerobacter mathranii*, similar genetic manipulations with an xylose-inducible promoter achieved ethanol production from xylose at 95% of the theoretical yield [77]. Expression of *Clostridium thermocellum* AdhE in *Caldicellulosiruptor bescii* led to ethanol formation at 33% of theoretical yield [78], while a heterologous expression of *Thermoanaerobacter* AdhA in *P. furiosus* enabled ethanol yields of 35% [79]. Moreover, co-expression of *P. furiosus* gene PF_0075, a homolog of Fe-AAdh from *T. neopolitana*, with AdhA (an NADP(H)-dependent primary ADH) or AdhE enhanced ethanol production, likely due to synergistic effects between Adh and Fe-AAdh encoded by PF_0075 [58,80]. These examples demonstrate that highly thermostable Fe-ADHs and related bifunctional ADHs can be harnessed to expand thermophilic biofuel production platforms.

Beyond biofuel applications, ADHs hold great promise as biocatalysts for the synthesis of enantiomerically pure alcohols as valuable intermediates in pharmaceutical and industrial applications [4]. ADHs from several extreme thermophiles and hyperthermophiles show a broad substrate specificity spectrum and produce exclusively (S)/(R)-enantiomer in high enantiomeric excess of greater than 90% [4]. Hyperthermophilic ADHs capable of reducing aromatic ketones to aromatic chiral alcohols are of major industrial interest due to their utility as building blocks in pharmaceutical synthesis, such as the short-chain ADH from *T. kodakarensis* [81] and the Zn-ADH from *Thermococcus guaymasensis* [82]. Although no Fe-ADHs from hyperthermophiles have yet been characterized with confirmed stereoselective activity toward chiral alcohol synthesis, the ADH from *T. hydrothermalis* exhibited substrate stereospecificity, oxidizing geraniol (trans) 7.5-fold more efficiently than its cis isomer, nerol [30]. This observation, combined with the large number of uncharacterized putative Fe-ADHs in hyperthermophilic genomes, suggests considerable potential to discover novel Fe-ADHs with high enantioselectivity and broad substrate acceptance.

The successful heterologous production of Fe-ADHs from hyperthermophiles in mesophilic hosts such as *E. coli* has significantly advanced their potential in industrial applications. The first recombinant Fe-ADH from *T. hydrothermalis* retained the thermophilicity, thermostability, and catalytic efficiency of the native enzyme when expressed in *E. coli* [30]. Remarkably, complete recovery of enzymatic activity was achieved simply by heat-induced refolding, demonstrating that fully active hyperthermophilic enzymes can be produced under mesophilic conditions [30]. Similarly, recombinant Fe-ADHs from *T. paralvinellae* ES-1 and *P. hypogea* were successfully expressed in *E. coli*, displaying biochemical properties indistinguishable from their native counterparts [27,36]. The recombinant *P. hypogea* ADH was also notably more resistant to oxidative inactivation than the native form [36]. It is also found that metal substitution at the active site, such as Ni^2+^-substitution in replacement of Fe^2+^ in the recombinant ADH of *P. horikoshii* OT3, enhanced oxygen tolerance while maintaining catalytic activity [34]. These findings highlight the feasibility of producing hyperthermophilic Fe-ADHs at the industrial scale and tailoring their stability through metal reconstitution or expression in mesophilic hosts for simplified cultivation, efficient purification, and higher protein yields.

Efficient cofactor regeneration is critical for the industrial application of hyperthermophilic ADHs in biocatalysis. The high cost of cofactors such as NAD(H) and NADP(H) necessitates the development of continuous regeneration systems to enhance economic viability. One widely explored strategy involves coupling ADHs with formate dehydrogenase (FDH) to regenerate NAD(P)H from NAD(P)^+^, using formate as a substrate to form the non-toxic byproduct CO_2_ [83,84]. In comparison to glucose dehydrogenase, this approach does not suffer from the formation of the byproduct gluconic acid, which requires additional pH adjustments to prevent acidification of the system [84]. Moreover, the majority of FDHs exhibit a broad pH range (6.0–9.0) or good thermostability (Tm ~ 60 °C) [84]. Among FDHs, metal-independent and oxygen-tolerant variants are particularly attractive for industrial applications [83]. Due to their versatility and potential in diverse bioprocesses, FDHs have become prominent targets for enzyme engineering [83]. Notably, mutants such as Phe285Thr/His311Gln and His311Gln of *Candida boidinii* FDH have demonstrated enhanced thermal stability, with melting temperatures of 73 °C and 77 °C, respectively, albeit with a trade-off in catalytic activity [85]. With the advent of advanced bioinformatics and protein engineering tools, the future of engineered FDHs appears promising, particularly for their integration with hyperthermophilic ADHs in robust cofactor regeneration systems.

To fully realize the industrial potential of Fe-ADHs from hyperthermophiles, challenges related to oxygen sensitivity and metal lability need to be addressed. Insights into these limitations have been provided by mutagenesis studies. In *E. coli*, single amino acid substitutions near the NAD-binding site (Ile7Leu, Leu8Val) in propanediol oxidoreductase increased resistance to oxidative stress at the cost of decreased thermal stability, while the Glu568Lys substitution in AdhE, another iron-containing ADH in *E. coli*, conferred aerobic functionality [86,87]. These findings suggest that similar approaches, such as site-directed mutagenesis of hyperthermophilic Fe-ADHs and metal substitution (e.g., Ni^2+^ for Fe^2+^), may yield more robust and oxygen-tolerant biocatalysts.

With ongoing advances in recombinant expression, structural analysis, and mutagenesis-guided design, Fe-ADHs from hyperthermophiles are poised to become indispensable catalysts for high-temperature biofuel production and stereoselective synthesis of chiral chemicals in industrial and pharmaceutical applications.

## 5. Conclusions

Fe-ADHs from hyperthermophiles exemplify a remarkable convergence of structural conservation and functional diversity shaped by extreme environmental adaptation. Phylogenetic analyses reveal several distinct clades, and within each clade, ADHs exhibit variations in cofactor specificity, substrate specificity, and kinetic properties. Despite these differences, all characterized Fe-ADHs retain the conserved Rossmann-fold cofactor-binding domain and iron-coordinating residues essential for redox catalysis. Their exceptional thermostability, often coupled with substrate variability, enables efficient catalysis at extremely high temperatures and under various physicochemical conditions. Functionally, most Fe-ADHs are predicted to contribute to aldehyde reduction and redox homeostasis, while some may also participate in hyperthermophilic fermentation pathways such as ethanol and butanol production. Beyond their physiological relevance, the robustness of these enzymes makes them attractive scaffolds for biotechnological applications.

## Figures and Tables

**Table 1:** Biophysical, biochemical, and kinetic properties of Fe-containing alcohol dehydrogenases from hyperthermophiles.

Organisms (Growth T_opt_ °C; Min–Max) ^a^	Sub- Unit (kDa)	Optimal Temperature ^b^ (°C)	Thermostability t_½_, h (at °C)	Substrate Specificity	Alcohol Oxidation				Aldehyde/Ketone Reduction				Ref.
					pH_opt_	App. *K*_m_ (mM) ^c^	App. *k*_cat_ (s^−1^) ^d^	App. *k*_cat_/ App. *K*_m_ (s^−1^ M^−1^)	pH_opt_	App. *K*_m_ (mM) ^e^	App. *k*_cat_ (s^−1^) ^d^	App. *k*_cat_/ App. *K*_m_ (s^−1^ M^−1^)	
*Thermococcus litoralis* (88; 55–98)	48 (α_4_)	80	2 (85) 0.25 (96)	1-hexanol, 1-pentanol, 1-butanol, 1-heptanol, 1-propanol, 1-octanol, isobutanol, ethanol, acetaldehyde	8.8	11 (ethanol) 0.033 (NADP)	26	2.4 × 10^3^ (ethanol)	N/A	0.4 (acetaldehyde) 0.1 (NADPH)	N/A	N/A	[19,25,39]
*Thermococcus paralvinellae* strain ES-1 (91; 82–91)	46 (α_4_)	˃95	35 (85) 4 (95)	1-hexanol, 1-pentanol, 1-butanol, 1-heptanol, 1-propanol, ethanol, isobutanol, 2-phenyl ethanol, 1-octanol, tryptophol, acetaldehyde, phenylacetaldehyde	8.8–10.4	8 (ethanol) 15 (2-phenylethanol) 6.3 (tryptophol) 0.014 (NADP)	48 (ethanol) 31.5 (2-phenylethanol) 17.6 (tryptophol) 41.9 (NADP)	6 × 10^3^ (ethanol) 2.1 × 10^3^ (2-phenylethanol) 2.8 × 10^3^ (tryptophol) 3 × 10^6^ (NADP)	7	0.25 (acetaldehyde) 0.05 (phenylacetaldehyde) 0.042 (NADPH)	19.1 (acetaldehyde) 15 (phenyl-acetaldehyde) 14.5 (NADPH)	7.6 × 10^4^ (acetaldehyde) 3 × 10^5^ (phenylacetaldehyde) 3.5 × 10^5^ (NADPH)	[26,27,40]
*Thermococcus* *zilligii* strain AN1 (75–80; 55–85)	46 (α_4_)	85	0.27 (80)	pentanol, butanol, ethanol, propanol, octanol	6.8–7.0	10 (ethanol) 12 (propanol) 1.4 (butanol) 0.08 (NADP)	N/A	N/A	N/A	0.12–0.13 (acetaldehyde)	N/A	N/A	[28,29,41]
*Thermococcus* *hydrothermalis* (85; 55–100)	45 (α_2_)	80	1.5 (80) (oxidation) 0.5 (80) 2.33 (70) (reduction)	aliphatic primary alcohols (C_2_–C_8_), benzyl alcohol, n-butanol, geraniol cyclohexanol, methanol, glycerol, nerol, benzaldehyde	10.5	2.0 (benzyl alcohol) 0.8 (NADP)	23 (benzyl alcohol) 25.6 (NADP)	1.2 × 10^4^ (benzyl alcohol) 3.2 × 10^4^ (NADP)	7.5	0.01 (benzaldehyde) 0.13 (NADPH)	1.7 (benzaldehyde) 61.1 (NADPH)	1.7 × 10^5^ (benzaldehyde) 4.7 × 10^5^ (NADPH)	[30,42]
*Thermococcus barophilus* Ch5 [Tba ADH_547_] (85; 48–100)	42	75	<0.5 (90) <0.5 (95) (reduction)	1-butanol, 1-hexanol, ethanol, ethylene glycol, isopentanol, isopropanol, glycerol, butyraldehyde, acetaldehyde, caproaldehyde, acetone	8.5	92 (ethanol)	0.27	2.9	7	34.5 (acetaldehyde)	69	2 × 10^3^	[31,43]
*Thermococcus barophilus* Ch5 [Tba ADH_641_] (85; 48–100)	44	~65 (oxidation) ~80 (reduction)	~0.33 (70) ~0.33 (85)	ethanol, 1-butanol, 1-hexanol, acetaldehyde, acetone, butylaldehyde, caproaldehyde	8	50 (ethanol)	7.2	140	8	1.5 (acetaldehyde)	40	2.7 × 10^4^	[32,43]
*Hyperthermus butylicus* (95–106; 80–108)	41	˃90	25 (95)	1-butanol, 2-butanol, 1-propanol, 1-pentanol, ethanol, 2-propanol, 2-phenylethanol, 1-hexanol, butyraldehyde, acetaldehyde, benzaldehyde, propanal, acetone, propanal	9	2.44 (butanol) 3.74 (2-propanol)	6.84 (butanol) 3.47 (2-propanol)	2.8 × 10^3^ (butanol) 928 (2-propanol)	6	0.59 (butyraldehyde) 0.68 (acetone)	8 (butyraldehyde) 3.84 (acetone)	1.4 × 10^4^ (butyraldehyde) 8.1 × 10^3^ (acetone)	[33,44]
*Pyrococcus horikoshii* OT3 (98; 85–105)	42	˃95 (reduction)	~6 (95)	1-butanol, 1-octanol, 1-heptanol, benzylalcohol, 1-hexanol, 1,4-butanediol, 1-pentanol, 1-propanol, ethanol, 1,3-propanediol, propanal, butanal, ethanal, linear aldehydes (C_1_, C_5_–C_8_)	9	0.52 (1-butanol) 0.03 (NAD)	0.48 (1-butanol) 0.50 (NAD)	920 (1-butanol) 2.1 × 10^4^ (NAD)	5.5	2.77 (propanal) 0.25 (butanal) 0.090 (pentanal) 0.011 (NADH)	7.87 (propanal) 6.96 (butanal) 6.66 (pentanal) 7.40 (NADH)	2.8 × 10^3^ (propanal) 2.8 × 10^4^ (butanal) 7.5 × 10^4^ (pentanal) 6.8 × 10^5^ (NADH)	[34,45]
*Pseudo-thermotoga hypogea* (70; 56–90)	40 (α_2_)	˃95	10 (70) 2 (90)	1-butanol, 1-propanol, 1-penanol, hexyl alcohol, 1-heptanol, 1-octanol, 1,5-pentanediol, 2-phenylethanol, ethanol, 1,4-butanediol, 1,3-propanediol, butyraldehyde, methylglyoxal, acetaldehyde	11	9.7 (ethanol) 1.9 (butanol) 0.020 (NADP)	14 (ethanol) 48 (butanol) 50 (NADP)	1.5 × 10^3^ (ethanol) 2.7 × 10^4^ (butanol) 2.5 × 10^6^ (NADP)	8	3.1 (acetaldehyde) 0.45 (butyraldehyde) 0.006 (NADPH)	15 (acetaldehyde) 48 (butyraldehyde) 52 (NADPH)	5 × 10^3^ (acetaldehyde) 1.1 × 10^5^ (butyraldehyde) 8.5 × 10^6^ (NADPH)	[35,36,46]
*Thermotoga neapolitana* [Fe-AAdh] (~80; 55–95)	43	95 (oxidation) 80 (reduction)	1 (92)	acetyl-coenzyme A, acetaldehyde, ethanol	8.82	N/A	N/A	N/A	7.54	1.75 (acetyl-CoA)	0.56 (acetyl-CoA)	320 (acetyl-CoA)	[37,47]

^a^ Optimal growth temperature (with minimum–maximum growth temperature range indicated); ^b^ optimal temperature for enzyme activity in alcohol oxidation, unless otherwise specified; ^c^ apparent *K*_m_ values, with the corresponding substrate (alcohol/cofactor) indicated in parentheses; ^d^ *k*_cat_ values were calculated from enzyme *V*_max_ and molecular weight reported in the reference publication(s), when not directly available; ^e^ apparent *K*_m_ values, with the corresponding substrate (aldehyde/ketone/cofactor/coenzyme) indicated in parentheses; N/A, not available. Data for native Fe-ADHs are presented, except where unavailable for *T. barophilus* Ch5 (ADH_547_ and ADH_641_), *P. horikoshii* OT3, and *T. neapolitana*, for which more detailed examinations were conducted in *T. hydrothermalis*, and for the optimum pH of reduction in *T. paralvinellae* strain ES-1; in such cases, data for recombinant forms are included.

**Table 2:** Predicted conserved cofactor- and iron-binding motifs of characterized Fe-ADHs from Hyperthermophiles.

Group ^1^	Characterized Fe-ADHs from Hyperthermophiles ^1^	Potential Cofactor-Binding Sequences and Their Positions ^1^		Potential Fe-Binding Sequences and Their Positions ^1^	
A	*T. zilligi* AN1, *T. hydrothermalis* and *T. barophilus* ADH_641_	GGGSPID	109, 110, 111, 112, 113, 114, 115	DALNH	Asp208, His212
				H, H, H, H	274, 277, 281, 292
	*T. paralvinellae* ES-1 and *T. litoralis* DSM	GGGSPID	110, 111, 112, 113, 114, 115, 116	DALNH	Asp209, His213
				H, H, H, H	275, 278, 282, 293
B	*P. hypogea*	GGGSVID	97, 98, 99,100, 101, 102, 103	DAIAH	Asp195, His199
				H, H, H	268, 272, 282
	*T. neapolitana*	GGGSPID	96, 97, 98, 99, 100, 101, 102	DAFYH	Asp196, His200
				H, H, H	265, 269, 280
C	*T. barophilus* ADH_547_	GGGSVID	92, 93, 94, 95, 96, 97, 98	DVLVH	Asp195, His199
				H, H, H, H	243, 262, 266, 274
	*P. horikoshii* OT3	GGGSVID	91, 92, 93, 94, 95, 96, 97	DVLVH	Asp194, His198
				H, H, H, H	242, 261, 265, 273
D	*H. butylicus*	GGGVSIE	64, 65, 66, 67, 68, 69, 70	H, E, H, H	130, 195, 223, 231

^1^ The table summarizes the predicted NAD(P)H-binding and Fe-binding sequences of characterized Fe-ADHs from hyperthermophiles, categorized into four groups (A–D) based on phylogenetic clustering analysis of hyperthermophilic Fe-ADHs. The potential cofactor-binding motifs correspond to the conserved glycine-rich region within the Rossmann fold, whereas the Fe-binding motifs include the conserved Asp and His residues/Glu and His residues coordinating the catalytic metal center. Amino acid positions are indicated according to the corresponding ADH sequences.

## Data Availability

The original contributions presented in this study are included in the article/Supplementary Material. Further inquiries can be directed to the corresponding author.

## Supplementary Materials

The following supporting information can be downloaded at: https://www.mdpi.com/article/10.3390/biotech15010006/s1, Table S1: NCBI accession numbers of Fe-ADHs from hyperthermophiles in Figure 2.
