# Supplementary material for: Iron-Containing Alcohol Dehydrogenase from Hyperthermophiles
Source: BioTech (Basel). 2026 Jan 15;15(1):6. doi: 10.3390/biotech15010006 (PMC12821551; doi:10.3390/biotech15010006)
Supplement: Supplementary file 1 [file biotech-15-00006-s001.zip › biotech-4030332-supplementary.pdf]

Review

Iron-Containing Alcohol Dehydrogenase from Hyperthermophiles

Ching Tse and Kesen Ma *

Department of Biology, University of Waterloo, Waterloo, ON, N2L 3G1 Canada; ching.tse@uwaterloo.ca
* Correspondence: kesen.ma@uwaterloo.ca

Table S1. NCBI accession numbers of Fe-ADHs from hyperthermophiles in Figure 2.

Hyperthermophiles	NCBI accession number
H. butylicus	WP_011822090.1
Palaeococcus pacificus DY20341	AIF69213.1
P. hypogea	AMT84600.1
Pyrococcus abyssi GE5	CAB50216.1
P. furiosus DSM 3638	AAC25557.1
P. horikoshii OT3	BAA29834.1
Pyrococcus kukulkanii	AMM53600.1
Thermococcus aciditolerans	QEK15767.1
Thermococcus aggregans	USS41611.1
Thermococcus alcaliphilus	MCO6041663.1
T. barophilus Ch5	ALM74514.1
T. barophilus Ch5 641	ALM74601.1
Thermococcus bergensis	MCA6214573.1
Thermococcus celericrescens	KUH32476.1
Thermococcus eurythermalis	AIU69195.1
T. hydrothermalis	CAA74334.1
Thermococcus indicus	QDA32374.1
Thermococcus kodakarensis	WCN27512.1
T. litoralis DSM 5473	EHR78112.2
Thermococcus onnurineus NA1	ACJ16031.1
Thermococcus pacificus	ASJ07663.1
T. paralvinellae ES-1	ACK56133.1
Thermococcus sibiricus MM 739	ACS90507.1
T. zilligii AN1	AAB63011.1
T. neapolitana DSM 4359	ACM22756.1
Thermotoga petrophila RKU-10	ADA66215.1
Thermotoga sp. RQ7	AJG39960.1
Thermotoga sp. SG1	PLV57609.1
